# Determination of Strain and Stress Field in Screening Test for Concrete Fire Spalling—Passive Restraint Effect

**DOI:** 10.3390/ma17246210

**Published:** 2024-12-19

**Authors:** Katarzyna Mróz, Izabela Hager, Marcin Tekieli, Václav Kočí, João Castro-Gomes

**Affiliations:** 1 Chair of Building Materials Engineering, Faculty of Civil Engineering, Cracow University of Technology, 24 Warszawska St., 31-155 Cracow, Poland; izabela.hager@pk.edu.pl; 2 Chair for Computational Engineering, Faculty of Civil Engineering, Cracow University of Technology, 24 Warszawska St., 31-155 Cracow, Poland; mtekieli@pk.edu.pl; 3 Department of Materials Engineering and Chemistry, Faculty of Civil Engineering, Czech Technical University in Prague, Thakurova 7/2077, 16629 Prague, Czech Republic; vaclav.koci@fsv.cvut.cz; 4 Centre of Materials and Building Technologies (C–MADE), Department of Civil Engineering and Architecture, University of Beira Interior (UBI), 6201-001 Covilhã, Portugal; jpcg@ubi.pt

**Keywords:** concrete, fire, spalling, cold rim, restraint, temperature, strain, stress, screening test

## Abstract

The paper examines the impact of passive restraint on fire-induced spalling in concrete, utilizing a concrete mixture to minimize compositional variability. A variety of specimen geometries was prepared, including standard cubes and cylinders for the determination of mechanical properties and slabs of different dimensions for fire spalling tests conducted under controlled conditions. A top-opening Dragon furnace, which applies ISO 834-1 fire curves, was used to evaluate the influence of “cold rim” boundaries, where slab edges were insulated to create thermal restraint. The cold rims were categorized as 0 cm, 10 cm, and 20 cm, with each modification representing a different degree of thermal expansion restraint. Digital image correlation (DIC) was utilized to monitor the strain fields on the unheated slab surfaces. The findings demonstrated that increasing the cold rim width implies a rise in compressive stress and strain in the central zone, thereby precipitating a more pronounced spalling behaviour. The unrestrained slabs (cold rim 0 cm) exhibited minimal spalling, whereas the restrained slabs (cold rim 20 cm) demonstrated significant spalling depths and volumes. The study confirms that thermal dilation restraint intensifies the severity of spalling and provides a quantitative framework that links stress evolution, strain distribution, and spalling depth. The findings emphasize the necessity of managing thermal restraint to properly assess fire-induced concrete spalling in material screening tests.

## 1. Introduction

So far, numerous experimental and numerical research was carried out to describe the problem of concrete fire spalling. A most common attempt is to indicate experimentally different parameters that may enhance spalling risk, such as the concrete properties, boundary conditions, etc. The variety of research setups and experimental methods has caused a wide diversity of obtained results [1,2,3], thus their comparison is difficult and clear conclusions are hardly drawn. The main objection to comparing spalling results from different laboratories is the influence of the materials and geometrical effects on spalling results. This was shown through an international comparison of screening test methods for the determination of fire spalling of concrete that was carried out among five laboratories [4]. The research summarized the results for different experimental stands, but “*no clear trend in spalling intensity*” was obtained. It confirmed the concerns about direct comparisons of the results and emphasized the need to standardize the test procedures for concrete fire spalling.

Recent concrete fire spalling experiments effectuated by a group of scientists indicated that concrete elements may experience self-restraint during fire [3,5,6,7,8,9]. It is believed that an unheated part of concrete (cold rim) may limit its thermal dilation and, hence, may increase the inner compressive stresses. In the case of a real fire event, the cold rim effect may occur in the unheated part of the structural element, for example, in the case of localized fire. In laboratory tests, such phenomenon may be reflected by the active or passive restraint of an investigated element.

Moreover, it was recently confirmed that there exists a link between the test configuration and the nature of the observed spalling during a fire test [10]. The form and intensity of spalling may result from different concrete mix compositions, moisture content, or its properties (for example, density, permeability, strength) but also may be affected by boundary conditions (i.e., fire scenario, support conditions). It was observed previously that also different stress states may involve various observations [11,12,13,14]. Corresponding to the cold rim effect, active restraint is taken into consideration by an externally applied load while passive restraint may be included by the unheated concrete edges or steel belt girdling the concrete element.

In the case of active restraint, the hydraulic jacks, a steel frame, bars, or tendons can be used to apply an in-plane compressive load. The load may be provided to reduce the thermal dilation of concrete or to induce compressive stress at a set level. The load level acting on the specimen is known and can be modulated during the tests. When comparing different mixes, active restraint tests guarantee that the same load is applied among the specimens.

In passive restraint tests, the compression in concrete is induced by a confining effect that can be provided by the concrete itself, pre-tensioning or post-tensioning tendons, a steel belt, or restraining frame. The cold rim does not experience thermal expansion and, hence, limits the thermal dilation of the heated central part of the specimen. Therefore, the cold rim creates a restraint to the inner part of the concrete and is believed to contribute to the development of compressive stresses in the heated part. When comparing different mixes, it should be emphasized that the in-plane load can differ among the different mixes.

In the last decades, the problem of a cold rim presence in a fire test has been discussed as the potential factor that may increase the concrete spalling sensitivity, and different conclusions have been drawn. The research program performed by [5] significantly showed that thermal stresses can be decisive for spalling to occur in concrete. In the experiment, 10 specimens were made of dense concretes of various compositions close to the one used for the Great Belt tunnel project [15]. The specimens were small slabs of 600 × 600 × 200 mm^3^ heated in a limited area of 200 × 200 mm^2^ at the centre of one side, so that the unheated part of the specimen around a fire-exposed area could resist the thermal expansion, according to what was described before. In all the tested specimens, progressive spalling was observed for about 20 min until the thermal stresses caused the cracking of the cold part. When the cracks occurred, spalling stopped immediately. It seems that, as soon as the internal thermal cracks were developed, the thermal compression stresses at the surface were released and, consequently, spalling was prevented unless the concrete external load or hindered thermal expansion of the entire cross-section caused additional stresses. For example, in the circular tunnel lining walls, the geometry itself causes the ongoing compressive stresses, and these restraints will be modelled if the fire tests are made on tunnel elements. The above research provides significant evidence for spalling susceptibility under a compressive load.

Next, the authors performed preliminary studies on the cold rim effect and tested three sizes of specimens heated in the same area with a torch [16]. As a result, the cold rim increased with larger specimens. Two types of concrete mixes were tested (gravel- and basalt-based concretes). The authors observed that, in tests on small samples without a cold rim, the release of water was facilitated and very well observed shortly after the start of fire exposure. In the cases of medium and big samples, the release of water through cracks was observed after an explosive spalling event. It was concluded that the thickness of the cold rim may affect the nature and the intensity of fire spalling in concrete. Moreover, it was raised that the cracking of the cold rim can limit the spalling event of a popcorn character but does not provide enough release of internal stresses and water pore pressure to prevent explosive spalling, as Hertz stated 15 years before [5].

In [17], the cracking of a cold rim was described after a fire test of seven slabs with different mixes. The intensity of the cracking corresponded well with the intensity of a spalling range. As the spalling became more intense, it generated higher internal tensile stresses that were distributed into the slab edging and resulted in the cracking of the cold rim.

Recently, the authors carried out an experimental program on the influence of different test configurations on a spalling nature and designed the sound-based method to indicate differences in spalling events that occur within different fire tests [10]. Within this study, the authors showed the differences in spalling natures and intensities of spalling events among concrete slabs with three ranges of a cold rim in the element. The tests confirmed that the wider a cold rim is, the strains in the concrete are greatest and the intensity of spalling increases. However, the paper does not provide an extensive comparison of the cold rim effect and its influence on fire test results. Hence, for this paper, the authors focused on the stress field in a concrete slab exposed to fire with different cold rim ratios and present its effectiveness in a screening test. The presented studies provide results that help us to understand how the unheated part of a slab may affect the spalling results in fire spalling screening tests. The latest RILEM recommendation on the method of testing concrete spalling due to fire—a material screening test [18]—highlights the importance of providing the restraining of concrete thermal dilation while testing. This paper explains this issue and proves the guidelines provided in the recommendations. In the following sections, the materials, methods, and results are presented to consider the passive restraint effect on concrete’s propensity for fire spalling.

## 2. Materials and Methods

To limit the influence of material composition, one concrete mix was assumed for this comparative study (Table 1). The decision on mix design was made according to a previous experimental campaign that tested 7 mixes made with cement and aggregate used widely in the Polish concrete market [19]. The chosen mix for the presentation in this paper was the mix that was prone to limited spalling at the unloading conditions (without passive restraint).

Different sizes of specimens were prepared for different purposes. For determining the initial and residual properties of concrete, standard cubic (150 mm × 150 mm × 150 mm) and cylindrical (Ø150 mm × H300 mm and Ø100 mm × H200 mm) specimens were manufactured. Also, for determining the stress–strain relation of loaded concrete, standard cylindrical specimens (Ø150 mm × H300 mm) were manufactured. For fire spalling tests in a furnace and the determination of the cold rim effect on spalling propensity, slab specimens of different geometries were produced. To provide three ranges of cold rim, three sizes of slabs were prepared, 600 × 600 × 150 mm^3^, 800 × 800 × 150 mm^3^, and 1000 × 1000 × 150 mm^3^. The summary of tested elements along with their dimensions and assigned purposes is presented in Table 2.

After moulding, the specimens were covered with a geotextile and watered every day for 14 days. After 14 days, the specimens were demoulded but, in the case of each element to be tested in a fire test, the bottom (fire-exposed) side was demoulded 14 days before running the fire test to prevent air drying. The cylinders for determining the moisture content were sealed accordingly to the concrete slab’s storage—sealed in the perimeter and at the bottom. The specimens were kept in a storage chamber with a relative humidity of 50 ± 5% until the day of the test. The age of the concrete specimen at the time of the fire test was between 300 and 330 days. The age of the concrete at the time of testing was chosen to limit the influence of concrete maturation processes and moisture content on the spalling results.

The concrete compressive strength at 28 days was 63.1 MPa, while at the time of testing (300 days), it was 67.85 MPa. The moisture content determined by drying the referenced sealed cylinders was 3.9% at the time of testing (300 days). Detailed results of the concrete properties are given in Table 3.

To provide the fire load, a Dragon furnace, presented in details in [20], was employed. The furnace has a top opening of 600 × 600 mm^2^ in size and is equipped with a 140 kW capacity gas burner. The temperature development in the furnace chamber refers to a standard fire scenario of ISO 834-1 [21]. For the test, the specimen was hung on the top of the furnace with a suspension system to ensure the exact position in the opening.

To analyse the cold rim effect, three ranges of cold rim were planned. As the furnace opening was unchanging, the fire-exposed area was the same for all the tested slabs. As a result of the different sizes of the concrete elements, three cold rim thicknesses were assumed:

- Cold rim 0 cm—all the bottom area was exposed to direct flames and no cold rim was present;
- Cold rim 10 cm—the 10 cm edges of the concrete slab were insulated with ceramic wool and did not experience direct heating;
- Cold rim 20 cm—the 20 cm edges of the concrete slab were insulated with ceramic wool and did not experience direct heating.

**Figure 1:**
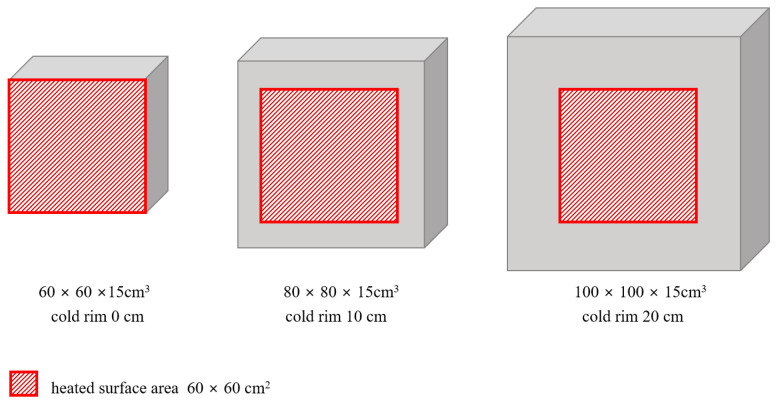
Scheme of tested elements.

The scheme of the above listed cases is presented in Figure 1.

**Figure 2:**
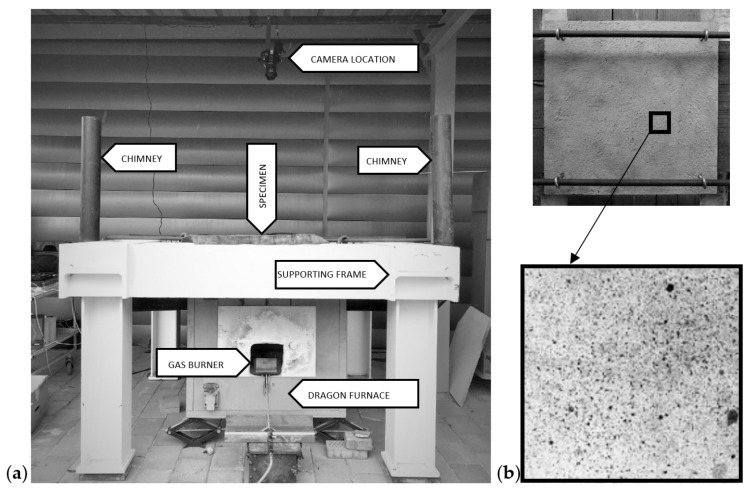
(**a**) Complete experimental setup, (**b**) black dots pattern for reference set.

To track the strain field in the element, the digital image correlation (DIC) method was employed. To take pictures of the unheated side for the DIC method, the camera was installed 1.5 m above the furnace on the steel substructure. During the fire load, pictures were taken every 10 s during the fire test by a DSLR Nikon D5300 camera (Nikon, Tokyo, Japan) with a matrix resolution of 24 Mpx (6000 × 4000 px) equipped with a low distortion zoom lens Sigma 17-50 f/2.8 EX DC OS HMS (Sigma, Warsaw, Poland). To create a subset of points to be tracked, randomly black dots were distributed over the concrete surface (Figure 2b). Having acquired pictures of unheated surfaces throughout the entire fire test, the images were pre-processed to, e.g., exclude noised pictures or equalize the colour space. Next, the DIC method was applied for the pre-processed images and the results were converted from a pixel coordination system to an object coordination system. Finally, the strain fields in the X, Y, and XY directions could be visualized in the form of a coloured map with a jet colour scale. The strain field was calculated for each picture taken during the test. Additionally, in post-processing, the DIC method enabled the placement of virtual extensometers at any point in the strain field and made it possible to define the reference distance. Based on the strains between two indicated ends of an extensometer, the strain development could be measured in the same way as real strain gauges. Thanks to the DIC method, the virtual extensometers could be added at any time and in any direction. This is undoubtedly one of the major advantages of using the DIC method in any kind of engineering test. To present the strain development in a time of fire exposure, virtual extensometers were used. The reference distance between the ends of the extensometers was equal to 50 mm. In the central zone, the virtual extensometers were distributed in the middle points of each side of the square rosette, of size 20 cm × 20 cm. The rosette was placed centrally in the middle of the examined specimen. In the case of placing the extensometers in the external zone, the same procedure was used. The size of the external rosette was adapted to the size of the slab individually.

**Figure 3:**
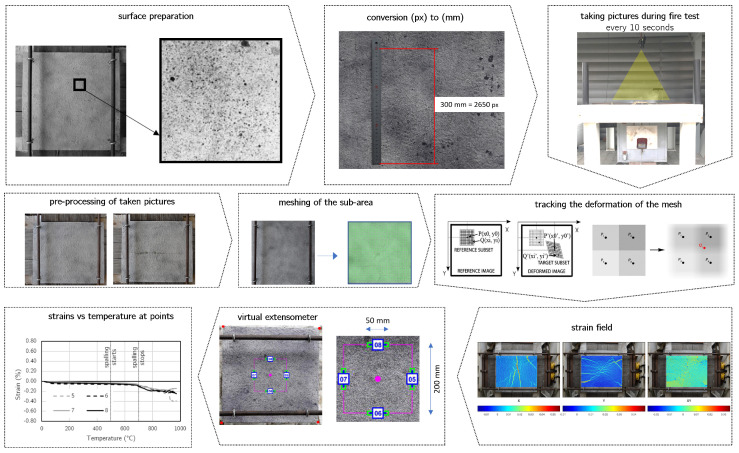
The order of testing procedure with the use of DIC method.

In Figure 2a, the complex setup with the Dragon furnace and camera location for DIC measurements is presented, while Figure 2b presents the pattern of black dots to be tracked by the DIC method. In Figure 3, the scheme of the testing procedure with the use of the DIC method is visualized. Before the fire test, the surface of the concrete element was covered with the black dots (surface preparation). Next, the camera was installed in front of the concrete element, from the unheated side, and the first picture was taken to obtain the conversion ratio. Based on the reference image, the equivalent of the specific metric length in pixels was known. Further, the fire test started and the pictures of the concrete surface were taken every 10 s. Having acquired the pictures of the surface throughout the entire test, the images were pre-processed to, e.g., exclude noised pictures or equalize the colour space. Next, the meshing of the area was performed and the images were converted from a pixel to an object coordination system. Thanks to the sub-pixel calculation, the accuracy of the DIC was here to 1/100 of the total pixel size. Next, the strain fields in the X, Y and XY directions were visualized as a coloured map. Finally, the virtual extensometers tracked the change in strains with temperatures at marked points.

**Figure 4:**
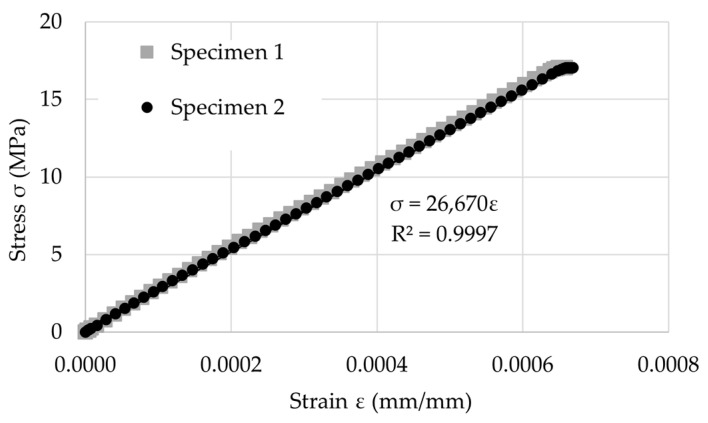
The stress–strain relationship and determination of the *σ*-*ε* formula.

To track the stress field on the top surface of the element, the preliminary loading test was performed on the 2 standard cylinders, Ø150 mm H300 mm, which were manufactured and cured in the same manner as the slabs for the fire tests. The cylinders were loaded until 17 MPa and the strains were monitored. As a result, the stress–strain (*σ*-*ε*) curves and formulas for this relationship were established. The diagram of the *σ*-*ε* relationship is presented in Figure 4. Having acquired the strain of the slab under fire conditions by virtual extensometers, Formula (1) was established to determine the stress field in the tested slabs.

(1) $$\sigma =26671\;\varepsilon \left(MPa\right),$$

where *σ* is the stress (MPa) and *ε* is the strain (mm/mm).

The slope of a linear Formula (1) reflects well the initial concrete modulus of elasticity (E = 26.67 GPa). As the top surface of the concrete slab remained cold during the time of the presented tests, it kept its elastic properties; thus, the calculations of stress development based on this linear correlation could be used.

## 3. Results

### 3.1. DIC Strain Fields

**Figure 5:**
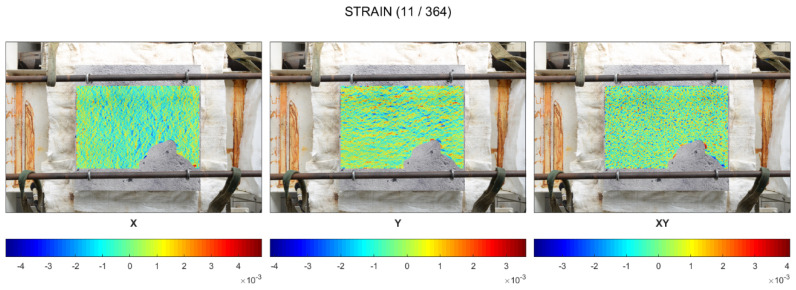
Cold rim 0 cm: maps of strains before the first event of spalling.

When the slab was exposed to fire on the entire bottom surface (cold rim 0 cm), the concrete did not experience restraint to thermal dilation due to the cold rim or the external load. As a result, in the very first minutes of fire exposure (up to ca. 2 min), before the first spalling event, the top surface did not present a significant strains distribution. In Figure 5, it is observed that the concrete worked in both directions and only local, random, higher strains indicated the localized stresses.

**Figure 6:**
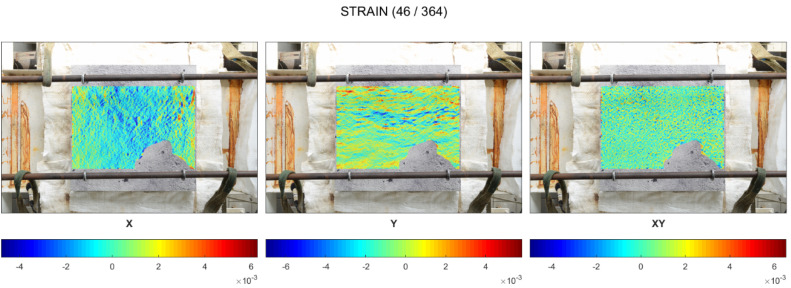
Cold rim 0 cm: maps of maximum strains during spalling occurrence.

From the time when the spalling started to occur (in the second minute of fire exposure), the top surface started to present compression (in blue) in the central part (Figure 6). Tension of the external edges was hardly noticed. While spalling events took place, no other significant observations were made. The strains of the top surface increased in time, but did not change the way of distribution.

To present the strain development in a time of fire exposure, virtual extensometers were used.

**Figure 7:**
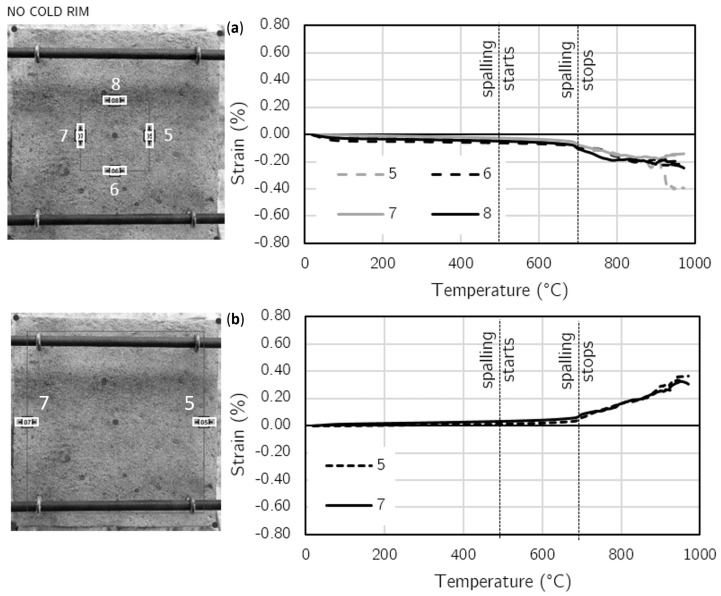
Cold rim 0 cm: strain development at fire-unexposed surface by virtual extensometers: (**a**) Strains in the central part of the unheated surface (X and Y direction), (**b**) Strains in most external part in X direction.

Figure 7 presents the strain development in the central zone of the unheated surface in the X and Y directions (Figure 7a) as well as strains in the external part in the X direction (Figure 7b). The virtual extensometers are indicated with numbers from 5 to 8, and the direction of measurement is designated as the white rectangle (the horizontal rectangle reflects the measurement in the X direction, while the vertical rectangle reflects the measurement in the Y direction).

Firstly, the diagrams of the strain development clearly indicate that the unrestrained slab worked in the symmetric condition during all the fire exposure time. Moreover, it can be observed that the spalling occurrence did not significantly influence the development of strain. During the spalling period, the strains changed from −0.045% to −0.103% in the central compression zone and from 0.032% to 0.068%. It confirmed that, if the concrete thermal dilations were not restrained, the stress state did not change significantly. Locally occurring spalling events were caused mainly by a thermal gradient in the layer directly exposed to the fire, and mainly aggregated spalling took place.

Moreover, the strains started to increase exactly when the spalling was terminated. It was probably directly linked to the extensive shrinkage of the drying concrete, as the water started to flow out in a liquid form, contributing to the acceleration of concrete drying.

**Figure 8:**
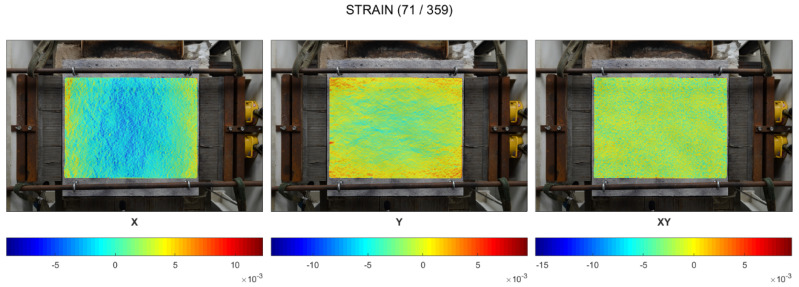
Cold rim 10 cm: maps of maximum strains during spalling events.

**Figure 9:**
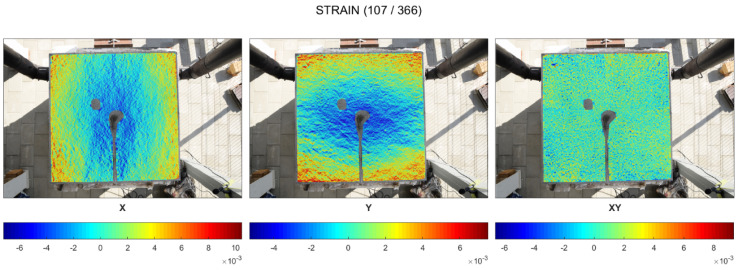
Cold rim 20 cm: maps of maximum strains during spalling events.

The maps of maximum strains in the time of spalling events for cold rim 10 cm and cold rim 20 cm are presented in Figure 8 and Figure 9, respectively. In the cases of testing the slab in which the thermal dilations were restrained by the cold rim (cold rim 10 cm and cold rim 20 cm), the strain distribution was like the unrestrained case, but its level increased significantly. There were no localized stresses, and the deformation was stable and uniform in the X and Y directions.

To present the strain distribution over the unheated surface, the virtual extensometers were employed at 10 points in the case of the 10 cm—cold rim and 12 points for the 20 cm—cold rim (four in the central part: two in the X direction and two in the Y direction; the others were in the external, cold rim zone).

**Figure 10:**
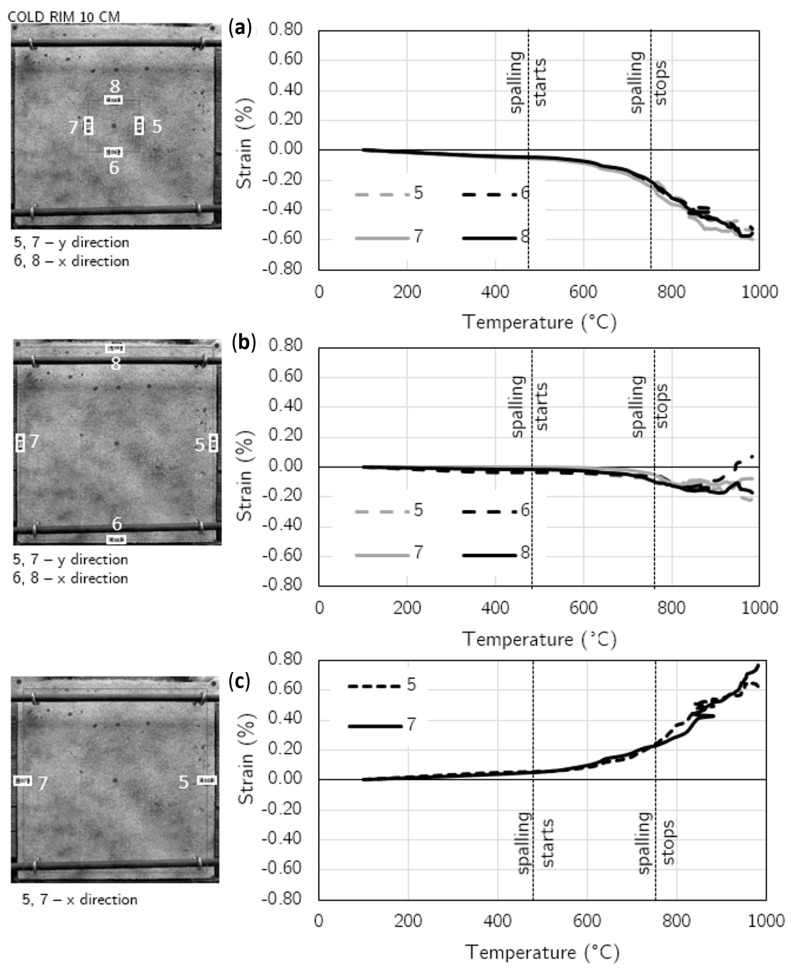
Cold rim 10 cm: strain development at fire-unexposed surface by virtual extensometers: (**a**) Strains in the central part of the unheated surface (X and Y direction), (**b**) Strains in most external part (cold rim) in X and Y direction; (**c**) Strains in most external part (cold rim) in X direction.

**Figure 11:**
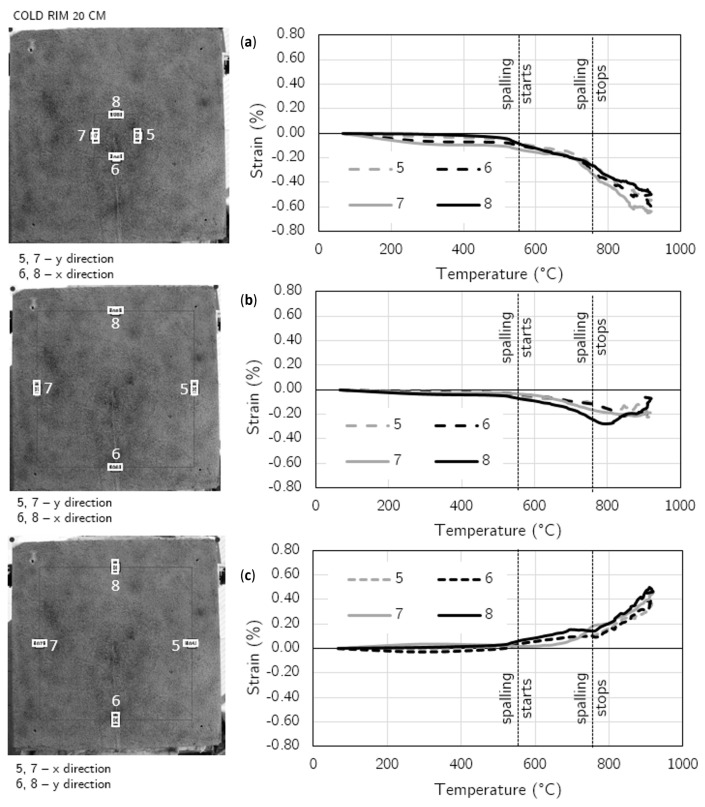
Cold rim 20 cm: strain development at fire-unexposed surface by virtual extensometers: (**a**) Strains in the central part of the unheated surface (X and Y direction), (**b**) Strains in most external part (cold rim) in X and Y direction; (**c**) Strains in most external part (cold rim) in X and Y direction.

In Figure 10, the diagrams of strain development in temperature are presented for each of the 10 virtual extensometers along with an indication of the placement of the virtual gauges for the slab restrained by a cold rim of 10 cm width. Figure 11 presents the results for the virtual extensometers for the slab restrained by a cold rim of 20 cm in width.

Until the first spalling event was noted, the slight compression in the central area was found with strains not exceeding −0.052% (cold rim 10 cm) and −0.091% (cold rim 20 cm) in compression and tension not exceeding 0.060% in the external edges in both the tested cases was noted. All those strains were symmetrical in both directions.

When the spalling was triggered (at 484 °C for the 10 cm cold rim and at 576 °C for the 20 cm cold rim), the strains increased in all direction. The compression in the central area was enhanced, while the tension of the cold rim increased as well. In the case of the cold rim of 10 cm, the highest strain in the compression central zone while spalling took place was measured as −0.208%, while for the cold rim of 20 cm, the maximum value reached −0.288%. It was due to the combined effect of thermal expansion of the concrete at a fire-exposed part and restraint to thermal dilation of the concrete by the cold rim that counteracted the concrete expansion and resulted in increasing the strains in compression.

Moreover, the effect of the cold rim could be analysed by the virtual extensometers placed at the outer part of the top surface. Based on the results presented in Figure 10c and Figure 11c, it can be clearly seen that the cold rim worked in tension during all the time of the fire exposure.

The restraint of thermal dilation by the cold rim of 20 cm in width caused higher strains in comparison to the cold rim of 10 cm. However, doubling the width of the unheated zone (cold rim) did not cause the great changes in the measured strains.

**Figure 12:**
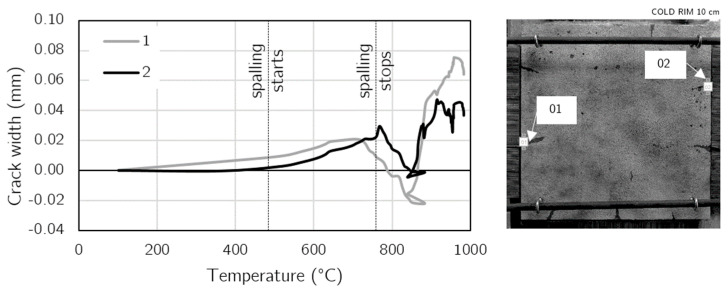
Development of crack width in the cold rim of 10 cm during fire exposure tracked by virtual extensometers.

The DIC method also enabled the installation of virtual extensometers in the post-processing stage. As the location of crack propagation on the unheated surface was well defined after the test, two virtual extensometers were used to track the development of the crack during fire exposure. Figure 12 presents the location of two virtual extensometers and the diagram of the development of crack width in the temperature domain. Some of the cracks started from the very beginning of fire exposure, while others seemed to propagate when the concrete started to spall. Next, the crack width increased until the spalling was limited. It was due to the continuous evaporation of water vapor through the cracks. When the water started to flow out in liquid form through the cracks, the spalling was terminated and the cracks started to close. Further, the crack again expanded over 800 °C due to the drying and intense concrete shrinkage.

### 3.2. Stress Development

The changes in stress that developed on the top surface of each tested slab were determined by Formula (1). The values of strains used for the calculation were taken from the virtual extensometers, presented in a previous paragraph. For each testing case (cold rim 0 cm, cold rim 10 cm, and cold rim 20 cm), the stress development was determined in the central zone of the unheated surface in the X and Y directions, in points presented in Figure 7a, Figure 10a and Figure 11a.

**Figure 13:**
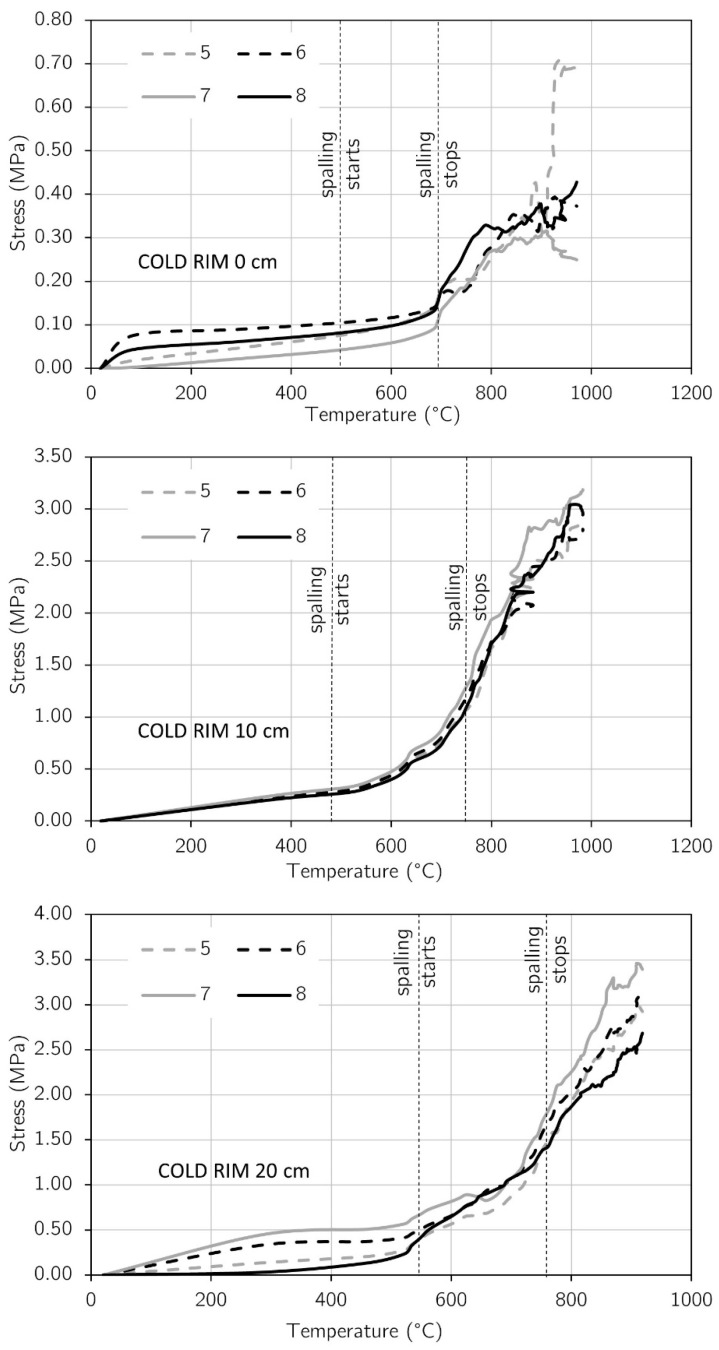
Development of stress in slabs during fire exposure.

In Figure 13, the results for stress development with temperature are presented for each testing case (cold rim 0 cm, cold rim 10 cm, and cold rim 20 cm). The diagrams show the clear differences among the tested panels. The stresses in the slab with the cold rim 0 cm were about 2 times lower than in the slab restrained with the 10 cm cold rim. Increasing the cold edge to 20 centimetres resulted in a visible increase in stress compared to the 10-centimetres restraint but not as abruptly as in comparison to the ‘cold rim 0 cm’ slab. Regardless of the stress values, the differences in the rate of stress development were observed while comparing with the spalling duration. As the ‘cold rim 0 cm’ slab was unrestrained, the spalling events did not influence the stress development. It seems that the spalling occurrence caused merely a cracking of the slab, and the stresses in the central part of the top surface increased slowly. However, as spalling stopped, the concrete started to release the liquid water through the already formed cracks. This accelerated the drying of the concrete, hence, its shrinkage and, as a result, a sharp increase in compressive stress.

On the other hand, in the case of the ‘cold rim 10 cm’ and ‘cold rim 20 cm’ slabs, the stress evolution started to accelerate just after the spalling began. It was clear evidence that, while the inner concrete was restrained, even with a slightly effective cold rim, the mismatch of the expansion of the inner concrete and nonexpanded cold rim caused growth in the compressive stresses that occurred in the top of the concrete slab. The continued increase in compressive stresses after the spalling termination was linked (similarly as for ‘cold rim 0 cm’) to the dynamic drying of the concrete. Evidence for such was the releasing of the liquid water through the cracks, as it was observed for the ‘cold rim 0 cm’ slab.

**Figure 14:**
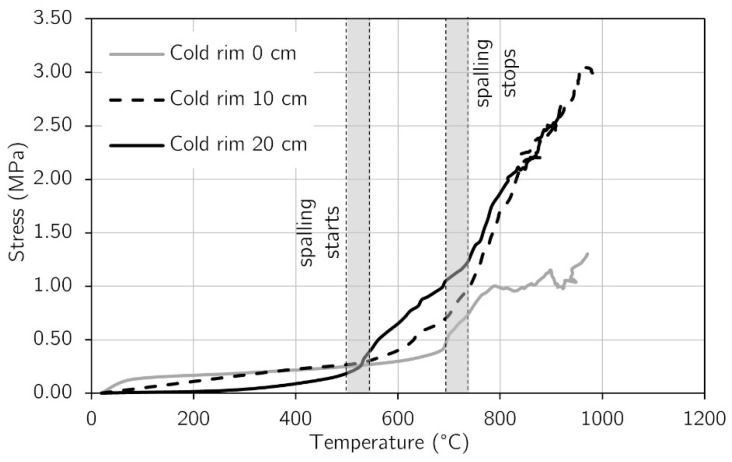
Comparison of stress evolution among testing cases.

To compare the results directly, the stress development in virtual extensometer 8 is compared in Figure 14. In the tested slabs, spalling started apparently in the same temperature, precisely between 484 °C and 576 °C, and terminated also with small differences in temperature (700 °C–743 °C) within all the tested slabs. Moreover, the same circumstances accompanied the termination of spalling events. The release of liquid water through the cracks took place and ended the spalling events. This suggests that the direct cause of spalling was the accumulation of water in the concrete cross-section and the associated significant pressure build-up in the pores of the material. As soon as the water found a way to escape, the internal pressure decreased and spalling stopped. However, in the discussed research, the differences appeared when spalling started. The internal pressure and different rates of restraint to concrete thermal dilation among slabs resulted in a changed dynamic of stress development. After the first spalling event (ca. 500 °C), the stresses in the ‘cold rim 10 cm’ and ‘cold rim 20 cm’ increased by ca. 1.75 and 2.60 times faster than the ‘cold rim 0 cm’, respectively. At the time of spalling end, the compressive stress reached 0.45 MPa for the ‘cold rim 0 cm’, 1.00 MPa for the ‘cold rim 10 cm’, and 1.43 MPa for the ‘cold rim 20 cm’. Such increase was directly affected by the limited capabilities for the water vapor and the liquid water release. The restraint with the 10 cm and 20 cm cold rims hindered the free cracking of the slab, as it took place in the ‘cold rim 0 cm’.

### 3.3. Spalling Results

After the fire test, the spalling depths and volume were measured. The results undeniably showed the differences between the levels of restraint for the unrestrained slab and those with a cold rim of 10 cm and 20 cm. In the ‘cold rim 0 cm’ and ‘cold rim 10 cm’, there were relatively small differences among the spalling depth D_max_ (10 mm and 16 mm), but in the ‘cold rim 20 cm’ slab, the spalling reached a depth of as much as 32 mm. In a fire situation, such a depth of damage reaches the reinforcement (thickness of cover) and begins to pose a threat to the stability of the structure, as it may lead to a situation in which the reinforcing steel is directly exposed to fire. The total spalling volume (V) also increased with the cold rim width. It should be remembered that the surface area of the slab that was directly exposed to fire in each of the three cases was the same. The volume of spalling in the ‘cold rim 20 cm’ was 4646 cm^3^, a value almost 20 times greater than in the unrestrained ‘cold rim 0 cm’ slab. As the research showed, increasing the compressive stresses in the slab caused greater spalling of the concrete, which confirms the hypothesis regarding the causes of spalling.

**Figure 15:**
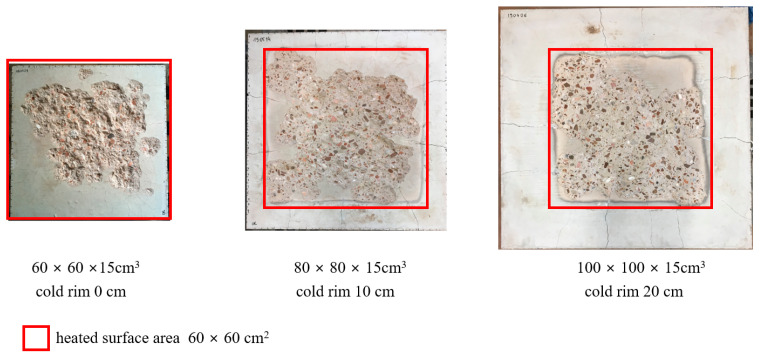
Damage range caused by spalling in tested slabs.

**Figure 16:**
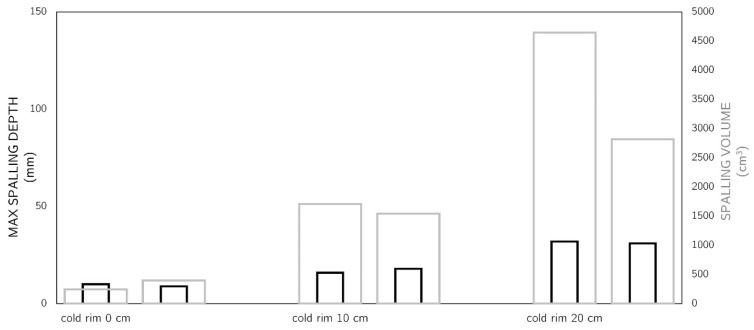
Comparison of measured max spalling depths and spalling volumes.

Figure 15 shows the pictures of the slabs after the fire tests and compares the damage range caused by the fire spalling of concrete, while Figure 16 presents the results of the spalling depths and volumes’ measurement.

Finally, Table 4 presents the principal results (mean from two slabs for each attempt) of the tests carried out within the presented research. This summary shows that the maximum values of strains and stresses directly influence the observations regarding spalling. It was proven that introducing any level of restraint to the thermal dilation of concrete during a fire event contributes significantly to its spalling propensity.

## 4. Discussion

During a fire, especially in its initial phase, the fire acts locally on the surface of the element, and the unheated, cooler part limits the thermal expansion of the concrete. In the tests of three types of concrete slabs, the generated stresses and deformations were determined by using the DIC image correlation method.

As the research showed, the DIC method allows placing virtual extensometers at any point in the deformation field and defining the reference distance. Based on the stresses between the two indicated ends of the virtual extensometer, the development of the strain can be measured in the same way as for a traditional extensometer. The accuracy of both methods is the same. Thanks to the DIC method, virtual strain gauges can be added at any time and in any direction. This is undoubtedly one of the main advantages of using the DIC method in all kinds of engineering research. Certainly, the digital image correlation method, thanks to its advantages, has the potential to be used in tests of concrete behaviour in a fire.

The extreme conditions of the test, driven by high temperatures and the unpredictable behaviour of the concrete surface exposed to fire, make the use of standard strain sensors infeasible on the heated side. Additionally, monitoring the deformation of nearly the entire surface is not achievable using any contact-based measurement methods. Therefore, the DIC (digital image correlation) method proved to be effective, providing novel insights in research on concrete spalling under fire exposure.

**Figure 17:**
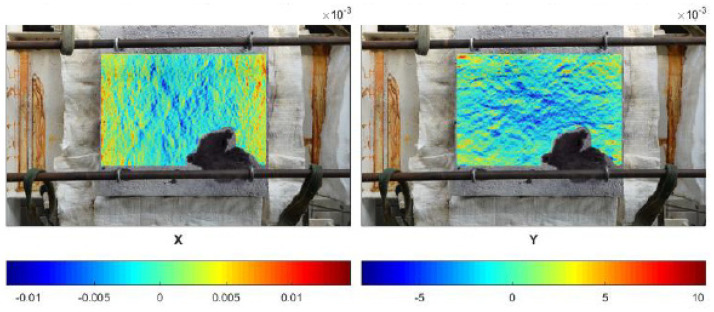
DIC limitation. The water accumulation on the specimen’s surface disrupts the DIC measurements.

During the tests, the DIC method encountered certain limitations. Studies confirmed that DIC measurements remain unaffected by water evaporation from the concrete specimen and that hot air does not interfere with obtaining reliable results. However, in some cases, a large volume of water is accumulated on the unexposed side, as shown in Figure 17. This water accumulation on the specimen’s surface significantly disrupts the DIC method’s ability to track pixels effectively during this period.

The research part presented the results of comparing the stresses and strains of heated concrete slabs with different levels of restraint. As shown by comparative studies, the level of thermal dilation restraint influenced both the stresses and deformations of concrete, consequently influencing the size and intensity of concrete spalling in a fire. The deformations and stresses occurring in the slabs with cold rims of 10 cm and of 20 cm were almost doubled compared to the slab with cold rim 0 cm. The most intense concrete spalling was observed in the slab restrained with a cold rim of 20 cm in width, for which the stresses reached 1.11 MPa during spalling events. The maximum spalling depth also increased with the level of restraint. Therefore, when considering the risk assessment of concrete fire spalling, it is recommended to use a test in which the passive restraint or a compressive load limits the crack opening. Such conditions are more unfavourable and better reflect the conditions in which spalling will arise.

The research also showed how much the extent of spalling during a fire is influenced by passive restraint with a cold rim. These observations relate to the local impact of fire and the risk of concrete spalling in the restrained element. The observed cracks and scratching of the cold edge led to a change in concrete stiffness in this zone. Parallelly, the closed cracks hindered water transport through the concrete elements toward a colder part and contributed to the developing of internal stresses. It may be concluded that a concrete element, which is heated over its entire surface during a fire and is free to deform, will show a lower tendency to spalling, while at the same time fulfilling the load-bearing capacity requirements for a longer time. However, such an impact of the fire on the element is realistically impossible, which excludes the determination of concrete fire behaviour in such a configuration.

This study presents findings that enhance the understanding of how the unheated portion of a slab can influence spalling outcomes in fire spalling screening tests. The latest RILEM recommendation on testing concrete spalling due to fire—the material screening test [18]—emphasizes the importance of restraining thermal dilation in specimens during testing. This paper addresses this issue and validates the assumptions outlined in the recommendations.

## 5. Conclusions

The conducted research on determining the influence of the level of restraint on the occurrence of the concrete spalling in fire contributes to a better understanding of the behaviour of concrete during a fire.

It is important to understand the impact of the test parameters on the test results of fire spalling of concrete. Such knowledge will enable the proper selection of test conditions as well as the selection of the size of samples/slabs and the method of loading them while testing. It is important to introduce a passive or active compressive stress in the concrete edge to prevent crack opening, as recommended in the current RILEM recommendations [18], so that the test conditions for the spalling behaviour of the concrete correspond to the more severe operating conditions of the heated element.

It is worth emphasizing that the tests aimed at determining the susceptibility of the material to spall should not be too complicated, and their implementation should not require advanced techniques (actuators, loading frames); perhaps the testing of slabs with a cold edge may be a simple solution for the initial comparison of fire behaviour of different materials in a fire condition.

In a further term, it seems necessary to conduct research on the concrete behaviour in a fire concerning, e.g., determining the level of compressive stresses on the intensity of fire spalling (thermal–mechanical mechanism).

It would also be interesting to test concrete slabs with polypropylene fibres and determine for what level of compressive stresses the fibres provide sufficient protection against spalling, allowing the water vapor pressure to be reduced after melting. Is there an amount of fibre that eliminates spalling in a fire, regardless of the stress level?

## Figures and Tables

**Table 1:** Mix component for tested concrete.

Component	Unit	Amount
CEM III A 42.5 N LH/HSR/NA (Górażdże, Poland)	kg/m^3^	400
Sand 0/2	kg/m^3^	640
Riverbed gravel 2/8	kg/m^3^	460
Riverbed gravel 8/16	kg/m^3^	690
Water	kg/m^3^	150
Admixture Atlas Primo LM-131 (Atlas, Rumia, Poland)	%mc	0.7
Admixture Hufgard AddiCRET PT20 (Hufgard, Czestochowa, Poland)	%mc	0.6

**Table 2:** Summary of manufactured elements.

Assigned Purpose		Dimensions (mm)		
Standard tests of properties	compressive strength	150	150	150
	modulus of elasticity	Ø150	300	-
	tensile strength	Ø100	200	-
	stress–strain test	Ø150	300	-
Fire tests	cold rim 0 cm	600	600	150
	cold rim 10 cm	800	800	150
	cold rim 20 cm	1000	1000	150

**Table 3:** Properties of tested concrete (at 28 days and at the time of testing = 300 days).

Density	Compressive Strength	Splitting Tensile Strength	Elastic Modulus	Fracture Energy	Mass Water Content	Compressive Strength
ρ (g/cm^3^)	f_c_ (MPa)	f_t.spl_ (MPa)	E (GPa)	G_f_ (J/m^2^)	w_m_ (%)	f_c_ (MPa)
at 28 Days	at 28 Days	at 28 Days	at 28 Days	at 28 Days	at 300 Days	at 300 Days
2.28	63.10	4.44	26.67	319.13	3.9	67.85

**Table 4:** Summary of stress, strain, and spalling results.

	T (°C) Temperature at First Spalling	*σ* (MPa) Max Stress	*ε* (mm/mm) Max Strain	D_max_ (mm) Max Spalling Depth	V (cm^3^) Spalling Volume
Cold rim 0 cm	539	0.69	0.0039	9.5	321.5
Cold rim 10 cm	484	1.05	0.0059	17.0	1628
Cold rim 20 cm	576	1.11	0.0061	31.5	3732

## Data Availability

The original contributions presented in this study are included in the article. Further inquiries can be directed to the corresponding author.
